# Synthesis and Antimicrobial Activity of Some New Pyrazoles, Fused Pyrazolo[3,4-*d*]-pyrimidine and 1,2-Dihydroimidazo-[2,1-*c*][1,2,4]triazin-6-one Derivatives

**DOI:** 10.3390/molecules16086549

**Published:** 2011-08-04

**Authors:** Sobhi Mohamed Gomha, Huwaida M.E. Hassaneen

**Affiliations:** Department of Chemistry, Faculty of Science, University of Cairo, Giza 12613, Egypt; Email: huwaidahassaneen@hotmail.com (H.M.E.H.)

**Keywords:** 2-hydrazinyl-5-imidazolone, hydrazonoyl halides, imidazo[2,1-*c*][1,2,4]triazinone, pyrazolo[3,4-*d*]pyrimidinone, pyrazoles, antimicrobial activity

## Abstract

A novel series of 7,7-diphenyl-1,2-dihydroimidazo[2,1-*c*][1,2,4]triazin-6(7*H*)-one **6a–h**, were easily prepared *via* reactions of novel 2-hydrazinyl-4,4-diphenyl-1*H*-imidazol-5(4*H*)-one (**2**) with hydrazonoyl halides **3a–h**. In addition, we also examined the reaction of compound **2** with commercially available active methylene compounds to afford new pyrazoles containing an imidazolone moiety, expected to be biologically active. The structures of the synthesized compounds were assigned on the basis of elemental analysis, IR, ^1^H-NMR and mass spectral data. The antifungal and antibacterial activities of the newly synthesized compounds were evaluated.

## 1. Introduction

Imidazoles are reported to have broad biological activities [1,2,3,4]. On the other hand, over the past two decades; pyrazole-containing compounds have received considerable attention owing to their diverse chemotherapeutic potential, including antineoplastic activities. Our literature survey revealed that some pyrazoles have been implemented as antileukemic [5,6], antitumor [7,8] and anti-proliferative [9] agents, in addition to their capability to exert remarkable anticancer effects through inhibiting different types of enzymes that play important roles in cell division [10]. Moreover, they have emerged as analgesic and anti-inflammatory drugs [11,12]. The synthesis of pyrazolo[3,4-*d*]-pyrimidine derivatives has also received significant attention in recent years because of their wide range of biological and pharmaceutical properties such as antitumor and antileukemia activity [13], anti-mycobacterial [14] and antidiabetic [15,16,17] agents, kinase [18,19] and phosphodiesterase [20] inhibitors, and also for their valuable antiangiogenic [21], fungicidal [22], cytotoxic [23] antitubercular [24], antimicrobial [25], potent antiproliferative agent [26] and anthelmintic [27] activities. In view of the above mentioned findings and as continuation of our effort [28,29,30,31] to identify new candidates that may be of value in designing new, potent, selective and less toxic antimicrobial agents, we report in the present work the synthesis of some new pyrazoles, pyrazolo[3,4-*d*]pyrimidine- and imidazo[2,1-c][1,2,4]triazinone derivatives starting from 2-hydrazinyl-4,4-diphenyl-1*H*-imidazol-5(4*H*)-one in order to investigate their antimicrobial activity.

## 2. Results and Discussion

**Scheme 1:**
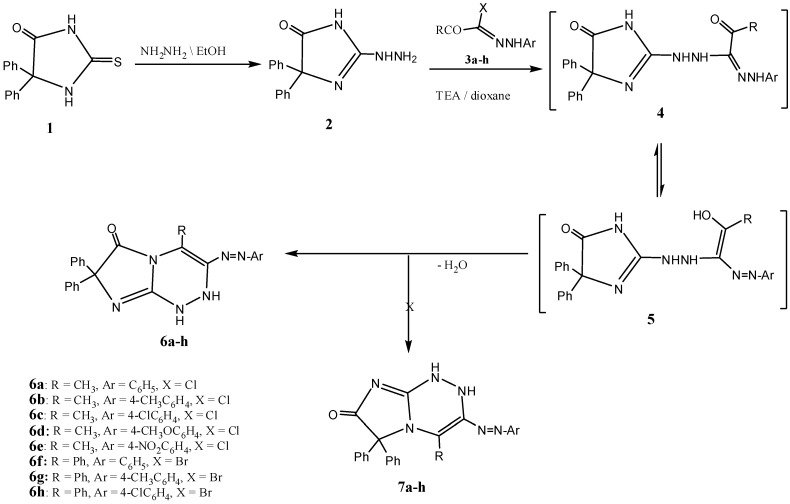
Synthesis of 3,4-disubstituted 7,7-diphenyl-1,2-dihydroimidazo[2,1-c]-[1,2,4]triazin-6(7*H*)-ones **6a–h**.

The required starting material 2-hydrazinyl-4,4-diphenyl-1*H*-imidazol-5(4*H*)-one (**2**) was prepared by reacting 5,5-diphenyl-2-thioxoimidazolidin-4-one (**1**) [32] with hydrazine hydrate in EtOH under reflux for 25 h (Scheme 1). The structure of **2** was elucidated on the basis of spectroscopic data and microanalysis. For example, its mass spectrum showed the correct molecular ion peak as a base peak. The IR spectrum of revealed typical absorption bands at 3440, 3324, 3228, 3166, 1724 cm^−^^1^ assignable to NH_2_, 2NH, and C=O moieties, respectively. The ^1^H-NMR spectrum showed a characteristic singlet signal at δ 2.10, assigned to the NH_2_ group.

Reaction of **2** with hydrazonoyl halides **3a–h** was carried in EtOH in the presence of triethylamine (TEA) and gave the corresponding substituted 7,7-diphenyl-3-(phenyldiazenyl)-1,2-dihydroimidazo[2,1-*c*][1,2,4]triazin-6(7*H*)-ones **6a–h** rather than the isomeric 4-substituted-6,6-diphenyl-3-(phenyldiazenyl)-1,2-dihydroimidazo[2,1-*c*][1,2,4]triazin-7(6*H*)-ones **7a–h** (Scheme 1).

**Figure 1:**
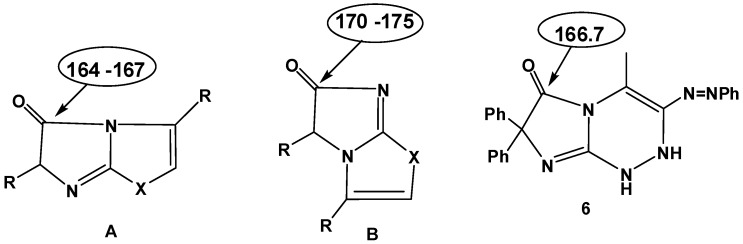
Comparison of C=O shifts with an *N*-atom in different bonding states next to C=O.

The structural elucidation of compounds **6a–h** was based on spectral evidence and microanalytical data. The mass spectra of them showed the molecular ion peaks at the expected *m/z* values. Their IR spectra indicated the disappearance of the NH_2_ group, and revealed in each case a C=O band in the region 1734–1710 cm^−1^ and two bands at 3430–3220 cm^−1^ assignable to 2NH groups. Also, their ^1^H-NMR spectra showed the presence of two signals for two NH groups at δ= 8.28–8.41 and 9.28–9.36 ppm. These two signals disappeared upon exchange with deuterium oxide. The ^13^C-NMR spectrum of **6a**, taken as an example for the series of compounds **6**, revealed a signal for the C=O group at δ= 166.7 ppm. This chemical shift value suggested that the *N*-1 near C=O is sp^3^ hybridized nitrogen atom pyrrole type, similar to that of compounds of type **A** (δ 164–167) and different from the sp^2^ hybridized nitrogen that of their isomers having structure **B** (δ 170–175) ppm (Figure 1). Based on the above finding we conclude that the isolated products have structures **6 **and not the isomeric structure **7**.

**Scheme 2:**
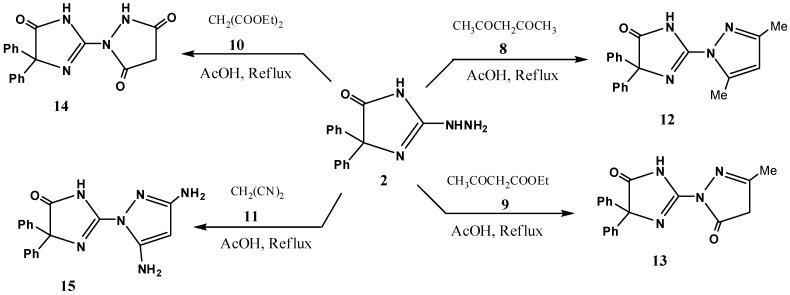
The reactivity of 2-hydrazino-4,4-diphenyl-1*H*-imidazol-5(4*H*)-one (**2**) towards active methylene reagents.

Finally, the suggestion that the site of cyclization of the intermediates **4** involves *N*-1 to give **6** is consistent with literature reports [33]. Our study was extended to the reaction of **2** with a variety of active methylene compounds, namely acetyl acetone (**8**), ethyl acetoacetate (**9**), diethyl malonate (**10**) and malononitrile (**11**) in order to synthesize compounds **12–15**, respectively (Scheme 2). These compounds have a pyrazole moiety and were anticipated to be biologically active. The structures of **12**–**15** were confirmed on the basis of spectroscopic data and elemental analyses (see Experimental section).

**Scheme 3:**
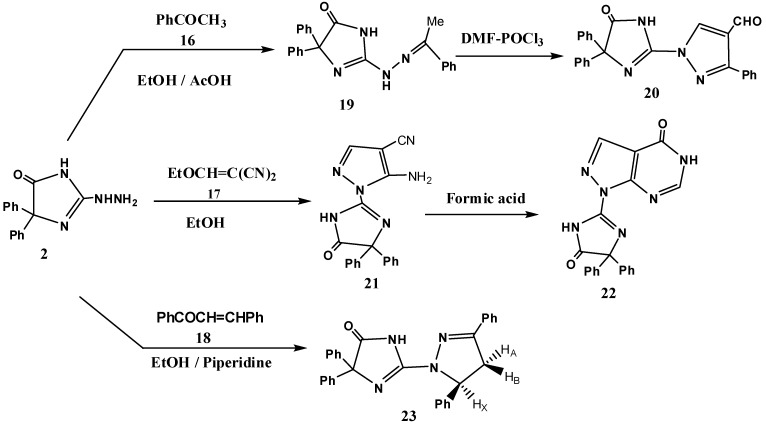
Synthesis of pyrazole derivatives from 2-hydrazino-4,4-diphenyl-1*H*-imidazol-5(4*H*)-one (**2**).

In addition, reaction of the hydrazine derivative **2** with acetophenone (**16**) gave the hydrazone **19**, which was converted further into the 1-(imidazol-2-yl) pyrazole-4-carbaldehyde **20** by treatment with Vilsmeier-Haack reagent (prepared by dropwise addition of phosphorus oxychloride in ice cooled DMF) [34]. The structure of the isolated aldehyde was confirmed on the basis of MS, IR, ^1^H-NMR spectra and elemental analysis. For example, the IR spectrum revealed absorption bands at 1681, 1724, 3166 cm^−1^ corresponding to 2 C=O and NH groups, respectively. The ^1^H-NMR spectra showed the presence of the NH and aldehyde groups at δ= 9.33, 9.88 ppm, respectively (Scheme 3). We also examined the reaction of **2** with ethoxymethylenemalononitrile (**17**). The isolated product was identified as the pyrazole derivative **21** on the basis of its elemental analysis and spectral data (Scheme 3). For example, the IR spectra of compounds **21** showed *v*_CN_ and *v*_CO_ near 2240 and 1724 cm^−1^, respectively (see Experimental section). The reaction of carbonitrile **21** with formic acid gave the corresponding 1-imidazol-2-yl-1*H*-pyrazolo[3,4-*d*]pyrimidin-4(5*H*)-one **22**. The lack of *v*_CN_ in the IR spectrum of the isolated product supported the formation of structure **22** (Scheme 3).

Furthermore, 1-(1*H*-pyrazol-1-yl)-1*H*-imidazol-5(4*H*)-one **23**, was prepared by reaction of **2** with chalcone **18** (Scheme 3). The structure of **23** was established based on its spectral data. The IR spectrum showed strong bands at 1722, 3169 cm^−1^ for C=O and NH, respectively. Also, the ^1^H-NMR spectra of **23** revealed no signal assignable to the NH_2_ group, while it revealed the presence of three characteristic signals due to the diasterotopic H atoms of a CH_2_ group coupled with H atom (Hx) next to it (HA, HB and HX). The HA proton which is *cis* to HX resonates upfield at δ 2.91 ppm as doublet of doublets (*dd*, *J* = 17.2 and 6.5 Hz), while HB which is *trans* to HX resonates downfield at δ 4.14 ppm (*dd*, *J* = 17.3 and 12.6 Hz). HX appeared as double of doublet at δ of 5.98 (*dd*, *J* = 12.8 and 6.5 Hz) (see Experimental section).

### Antimicrobial Activity

The compounds were tested for their activities against Gram +ve bacteria (*Staphylcoccus aureus*) and Gram –ve bacteria (*Escherichia coli*), in addition to the pathogenic fungi *Aspergillus flavus* and *Candida albicans*. The antimicrobial screening results were measured by the average diameter of the inhibition zones, expressed in mm, and are depicted in Table 1. The results showed that, all the tested compounds displayed significant activities against *E. coli* and *S. aureus*, while, only compounds **6c**, **6h** and **20** were moderately active against *A. flavus* and *C. albicans*. However, the activities of the tested compounds are much less than those of standard antifungal and antibacterial agents used.

**Table 1:** Antimicrobial activity of the tested compounds.

Sample No.	Inhibition zone diameter (mm/mg sample)			
	*E. coli* (G^−^)	*S. aureus* (G^+^)	Fungus	
			*A. flavus*	*C. albicans*
**6a**	22	16	--	--
**6c**	21	20	10	15
**6f**	14	16	--	--
**6h**	15	13	9	13
**12**	18	15	--	--
**13**	24	12	--	--
**14**	16	19	--	--
**15**	18	14	--	--
**20**	12	18	11	14
**22**	18	22	--	--
**23**	14	23	--	--
Tetracycline	30	30	--	--
Amphotricine	--	--	18	21

* The concentration of the solution 20.0 mg/mL was tested; *E. coli*: *Esherichia coli*; G^−^: Gram negative bacteria; *S. aureus*: *Staphylococcus aureus*; G^+^: Gram positive bacteria; *A. flavus*: *Aspergillus flavus*; *C. albicans*: *Candida albicans*.

## 3. Experimental

### 3.1. General

All melting points were measured on Electrothermal IA 9000 series digital melting point apparatus. The IR spectra were recorded in potassium bromide discs on a Pye Unicam SP 3300 or Shimadzu FT IR 8101 PC infrared spectrophotometers. The NMR spectra were recorded at 270 MHz on a Varian Mercury VX-300 NMR spectrometer. ^1^H-NMR (300 MHz) and ^13^C-NMR (75.46 MHz) were run in deuterated chloroform (CDCl_3_) or dimethylsulphoxide (DMSO-*d*_6_). Chemical shifts were related to those of the solvent. Mass spectra were recorded on a Shimadzu GCMS-QP1000 EX mass spectrometer at 70 eV. Elemental analyses and the biological evaluation of the products were carried out at the Microanalytical Centre of Cairo University, Giza, Egypt. All reactions were followed by TLC (Silica gel, Aluminum Sheets 60 F254, Merck). Hydrazonoyl chlorides **3a–g**[35,36] were prepared as reported in the literature.

### 3.2. 2-Hydrazinyl-4,4-diphenyl-1H-imidazol-5(4*H*)-one *(**2**)*

To 2-thioxoimidazolidin-4-one 1 (1.0 g, 4 mmol) in dry EtOH (10 mL) was added hydrazine hydrate (80%, 2 mL). The reaction mixture was kept under reflux for 25 h, and then cooled. The solid which precipitated was filtered off and crystallized from DMF to give **2** in 70% yield, m.p. 354 °C; MS *m/z* (%): 266 (M^+^, 70), 248 (45), 165 (42), 104 (35), 77 (100), 66 (33); IR (KBr): *v* 3440, 3324 (NH_2_), 3228, 3166 (2NH), 1724 (CO) cm^−1^; ^1^H-NMR (CDCl_3_): d 2.10 (s, 2H, NH_2_), 3.57 (s, 1H, NH), 7.34–8.23 (m, 10H, Ar–H), 9.34 (s, 1H, NH); Anal. Calcd. for C_15_H_14_N_4_O (266.12): C, 67.65; H, 5.30; N, 21.04%. Found: C, 67.3; H, 5.32; N, 21.21%.

### 3.3. 3,4-Disubstituted 7,7-diphenyl-1,2-dihydroimidazo[2,1-c][1,2,4]triazin-6(7*H*)-ones ***6a–h***

General procedure: To **2** (2.68 g, 10 mmol) and the appropriate hydrazonoyl halides **3a–h** (10 mmol) in dioxane (50 mL) was added triethylamine (1.4 mL, 10 mmol) at room temperature. The reaction mixture was heated under reflux until all the starting material was consumed (6–10 h, monitored by TLC). The solvent was evaporated and the residue was triturated with MeOH. The formed solid was filtered and recrystallized from DMF to give compounds **6a–h**.

*4-Methyl-7,7-diphenyl-3-(phenyldiazenyl)-1,2-dihydroimidazo[2,1-c][1,2,4]triazin-6(7*H*)-one* (**6a**). Yield 82%; red crystals (from EtOH); m.p. 152 °C; IR (KBr): *v* 1724 (C=O), 3425, 3259 (2NH) cm^−1^; ^1^H-NMR (DMSO-*d*_6_): δ 2.53 (s, 3H, CH_3_), 6.98–7.52 (m, 15H, Ar–H), 8.31 (s, 1H, NH), 9.36 (s, 1H, NH); MS *m/z* (%): 408 (M^+^, 6), 248 (13), 206 (29), 165 (20), 91 (30), 77 (100), 51 (53); ^13^C-NMR (DMSO-*d*_6_) δ ppm: 166.7 (C=O), 158.6 (C=N), 147.3, 139.8, 139.6, 133.8, 132.4, 132.1, 130.9, 130.8, 127.8, 127.1, 124.7, 121.9 (Ar–C) 118.6, 114.3 (C=C), 71.3 (Ph_2_C), 8.4 (CH_3_); Anal. Calcd for C_24_H_20_N_6_O (408.17): C, 70.57; H, 4,94; N, 20.58%. Found: C, 70.54; H, 4.88; N, 20.50%.

*4-Methyl-7,7-diphenyl-3-(p-tolyldiazenyl)-1,2-dihydroimidazo[2,1-c][1,2,4]triazin-6(7*H*)-one* (**6b**). Yield 80%, red crystals (from EtOH), m.p. 164 °C; IR (KBr): *v* 1724 (C=O), 3425, 3257 (2NH) cm^−1^; ^1^H-NMR (DMSO-*d*_6_): δ 2.25 (s, 3H, CH_3_), 2.52 (s, 3H, CH_3_), 6.96–7.48 (m, 10H, Ar–H), 7.54 (d, *J* = 7.2 Hz, 2H, Ar–H), 8.14 (d, *J* = 7.2 Hz, 2H, Ar–H), 8.31 (s, 1H, NH), 9.30 (s, 1H, NH); MS *m/z* (%): 422 (M^+^, 6), 341 (72), 299 (11), 165 (66), 91 (85), 77 (100), 52 (30); Anal. Calcd for C_25_H_22_N_6_O (422.19): C, 70.07; H, 5.25; N, 19.89%. Found: C, 70.04; H, 5.22; N, 19.74%.

*3-[(4-Chlorophenyl)diazeny]-4-methyl-7,7-diphenyl-1,2-dihydroimidazo[2,1-c][1,2,4]triazin-6(7*H*)-one* (**6c**). Yield 85%, red crystals (from EtOH), m.p. 136 °C; IR (KBr): *v* 1722 C=O), 3423, 3254 (2NH) cm^−1^; ^1^H-NMR (DMSO-*d*_6_): δ 2.53 (s, 3H, CH_3_), 6.99–7.53 (m, 10H, Ar–H), 7.58 (d, *J* = 8.4 Hz, 2H, Ar–H), 8.22 (d, *J* = 8.4 Hz, 2H, Ar–H), 8.28 (s, 1H, NH), 9.30 (s, 1H, NH); MS *m/z* (%): 442 (M^+^, 26), 401 (32), 360 (19), 165 (57), 91 (13), 77 (100), 51 (60); Anal. Calcd for C_24_H_19_ClN_6_O (442.13): C, 65.08; H, 4.32; N, 18.97%. Found: C, 65.04; H, 4.30; N, 18.74%.

*3-[(4-Methoxyphenyl)diazeny]-4-methyl-7,7-diphenyl-1,2-dihydroimidazo[2,1-c][1,2,4]triazin-6(7*H*)-one* (**6d**). Yield 79%, red crystals (from EtOH), m.p. 122 °C; IR (KBr): *v* 1724 (C=O), 3422, 3253 (2NH) cm^−1^; ^1^H-NMR (DMSO-*d*_6_): δ 2.51 (s, 3H, CH_3_), *δ* 3.36 (s, 3H, CH_3_), 6.97–7.52 (m, 10H, Ar–H), 7.58 (d, *J* = 8.0 Hz, 2H, Ar–H), 8.21 (d, *J* = 8.0 Hz, 2H, Ar–H), 8.28 (s, 1H, NH), 9.32 (s, 1H, NH); MS *m/z* (%): 439 (M^+^, 14), 338 (28), 208 (27), 165 (100), 91 (42), 77 (100), 51 (30). Anal. Calcd for C_25_H_22_N_6_O_2_ (438.18): C, 68.48; H, 5.06; N, 19.17%. Found: C, 68.44; H, 5.02; N, 19.12%.

*4-Methyl-3-[(4-nitrophenyl)diazenyl]-7,7-diphenyl-1,2-dihydroimidazo[2,1-c][1,2,4]triazin-6(7*H*)-one* (**6e**). Yield 77%, red crystals (from EtOH), m.p. 146 °C; IR (KBr): *v* 1723 (C=O), 3425, 3255 (2NH) cm^−1^; ^1^H-NMR (DMSO-*d*_6_): δ 2.5 (s, 3H, CH_3_), 6.97–7.55 (m, 10H, Ar–H), 7.58 (d, *J* = 7.2 Hz, 2H, Ar–H), 8.24 (d, *J* = 7.2 Hz, 2H, Ar–H), 8.29 (s, 1H, NH), 9.36 (s, 1H, NH); MS *m/z* (%): 453 (M^+^, 15), 372 (40), 248 (23), 180 (100), 165 (32), 104 (68), 77 (76), 51 (49); Anal. Calcd for C_24_H_19_N_7_O_3_ (453.15): C, 63.57; H, 4.22; N, 21.62%. Found: C, 63.54; H, 4.20; N, 21.58%.

*4,7,7-Triphenyl-3-(phenyldiazenyl)-1,2-dihydroimidazo[2,1-c][1,2,4]triazin-6(7*H*)-one* (**6f**). Yield 80%, red crystals (from EtOH), m.p. 118 °C; IR (KBr): *v* 1732 (C=O), 3425, 3264 (2NH) cm^−1^; ^1^H-NMR (DMSO-*d*_6_): δ 6.91–7.59 (m, 20H, Ar–H), 8.41 (s, 1H, NH), 9.28 (s, 1H, NH); MS *m/z* (%): 470 (M^+^, 11), 326 (21), 297 (31), 165 (74), 91 (38), 76 (100), 52 (44); ^13^C-NMR (DMSO-*d*_6_) δ ppm: 166.7 (C=O), 158.2 (C=N), 147.1, 140.4, 139.3, 139.2, 133.8, 133.2, 133.0, 132.1, 130.9, 130.8, 127.8, 127.1, 124.7, 124.3, 121.1, 120.9 (Ar–C) 118, 114 (C=C), 71.3 (Ph_2_C); Anal. Calcd for C_29_H_22_N_6_O (470.19): C, 74.03; H, 4.71; N, 17.86%. Found: C, 70.04; H, 4.58; N, 17.63%.

*4,7,7-Triphenyl-3-(p-tolyldiazenyl)-1,2-dihydroimidazo[2,1-c][1,2,4]triazin-6(7*H*)-one* (**6g**). Yield 78%, red crystals (from EtOH), m.p. 124 °C; IR (KBr): *v* 1730 (C=O), 3425, 3266 (2NH) cm^−1^. ^1^H-NMR (DMSO-*d*_6_): δ 2.27 (s, 3H, CH_3_), 6.94–7.55 (m, 15H, Ar–H), 7.57 (d, *J* = 7.2 Hz, 2H, Ar–H), 8.20 (d, *J* = 7.2 Hz, 2H, Ar–H), 8.40 (s, 1H, NH), 9.28 (s, 1H, NH); MS *m/z* (%): 484 (M^+^, 18), 329 (26), 284 (18), 165 (57), 91 (32), 76 (100), 52 (54); Anal. Calcd for C_30_H_24_N_6_O (484.20): C, 74.63; H, 4.99; N, 17.34%. Found: C, 74.71; H, 4.87; N, 17.21%.

*3-[(4-Chlorophenyl)diazenyl]-4,7,7-triphenyl-1,2-dihydroimidazo[2,1-c][1,2,4]triazin-6(7*H*)-one* (**6h**). Yield 81%, red crystals (from EtOH), m.p. 142 °C; IR (KBr): *v* 1732 (C=O), 3424, 3246 (2NH) cm^−1^; ^1^H-NMR (DMSO-*d*_6_): δ 6.94–7.55 (m, 15H, Ar–H), 7.64 (d, *J* = 8.1 Hz, 2H, Ar–H), 8.24 (d, *J* = 8.1 Hz, 2H, Ar–H), 8.41 (s, 1H, NH), 9.31 (s, 1H, NH); MS *m/z* (%): 504 (M^+^, 18), 326 (25), 165 (100), 91 (41), 77 (65), 52 (37); Anal. Calcd for C_29_H_21_Cl N_6_O (504.15): C, 68.98; H, 4.19; N, 16.64%. Found: C, 68.90; H, 4.11; N, 16.60%.

### 3.4. Reaction of ***2*** with Active Methylene Compounds

General procedure: A mixture of compound **2** (1.34 g, 5 mmol) and active methylene compound (5 mmol) in glacial acetic acid (20 mL) was refluxed for 6 h. After cooling, the precipitate was collected by filtration and crystallized from the appropriate solvent to afford compounds **12–15**.

*2-(3,5-Dimethyl-1H-pyrazol-1-yl)-4,4-diphenyl-1*H*-imidazol-5(4*H*)-one* (**12**). Yield 80%, Pale yellow solid (from EtOH), m.p. 220 °C; IR (KBr): *v* 1724 (C=O), 3166 (NH) cm^−1^; ^1^H-NMR (DMSO-*d*_6_): δ 2.32 (s, 3H, CH_3_), 2.49 (s, 3H, CH_3_), 6.08 (s, 1H, pyrazolyl–H), 7.12–7.98 (m, 10H, Ar–H), 9.33 (s, 1H, NH); MS *m/z* (%): 330 (M^+^, 30), 223 (41), 180 (100), 104 (51), 77 (53), 51 (49); Anal. Calcd for C_20_H_18_ClN_4_O (330.15): C, 72.71; H, 5.49; N, 16.96%. Found: C, 72.68; H, 5.44; N, 16.86%.

*3-Methyl-1-(5-oxo-4,4-diphenyl-4,5-dihydro-1*H*-imidazol-2-yl)-1H-pyrazol-5(4*H*)-one* (**13**). Yield 82%, Pale yellow micro-crystals (from EtOH), m.p. 266 °C; IR (KBr): *v* 1678, 1690, 1720 (3C=O), 3169 (NH) cm^−1^; ^1^H-NMR (DMSO-*d*_6_): δ 2.41 (s, 3H, CH_3_), 3.52 (s, 2H, CH_2_), 7.12–7.96 (m, 10H, Ar–H), 9.33 (s, 1H, NH); MS *m/z* (%): 332 (M^+^, 22), 223 (41), 180 (100), 104 (47), 77 (33), 51 (58); Anal. Calcd for C_19_H_16_Cl N_4_O_2_ (332.13): C, 68.66; H, 4.85; N, 16.86%. Found: C, 68.85; H, 4.79; N, 16.61%.

*1-(4-Oxo-4,4-diphenyl-4,5-dihydro-1*H*-imidazol-2-yl)pyrazolidine-3,5-dione* (**14**). Yield 74%, yellow crystals (from EtOH-dioxane), m.p.292 °C; IR (KBr): *v* 1679, 1690, 1724 (3C=O), 3166, 3210 (2NH) cm^−1^; ^1^H-NMR (DMSO-*d*_6_): δ 4.82 (s, 2H, CH_2_), 7.12–7.98 (m, 10H, Ar–H), 9.33 (s, 1H, NH), 10.64 (s, 1H, NH); MS *m/z* (%): 334 (M^+^, 23), 223 (31), 180 (64), 104 (33), 77 (100), 51 (42); Anal. Calcd for C_18_H_14_N_4_O_3_ (334.11): C, 64.66; H, 4.22; N, 16.76%. Found: C, 64.54; H, 4.12; N, 16.69%.

*2-(3,5-Diamino-1*H*-pyrazol-1-yl)-4,4-diphenyl-1H-imidazol-5(4*H*)-one* (**15**). Yield 80%, Pale yellow solid (from EtOH), m.p. 312 °C; IR (KBr): *v* 1722 (C=O), 3166 (NH), 3212–3360 (2NH_2_) cm^−1^; ^1^H-NMR (DMSO-*d*_6_): δ 6.01 (s, 1H, pyrazolyl–H), 6.34–6.48 (s, 4H, 2NH_2_), 7.12–7.98 (m, 10H, Ar–H), 9.33 (s, 1H, NH); MS *m/z* (%): 332 (M^+^, 30), 223 (41), 180 (100), 104 (51), 77 (53), 51 (49); Anal. Calcd for C_18_H_16_N_6_O (332.14): C, 65.05; H, 4.85; N, 25.29%. Found: C, 65.10; H, 4.68; N, 25.21%.

*Synthesis of 4,4-diphenyl-2-(2-(1-phenylethylidene)hydrazinyl)-1*H*-imidazol-5(4*H*)-one* (**19**). A mixture of **2** (2.68 g, 10 mmol) and acetophenone **16** (1.20 g, 10 mmol) in 20 mL absolute ethanol was refluxed in water bath for 4 h in presence of glacial acetic acid (1 mL). The product obtained after cooling was crystallized from absolute ethanol. Yield 80%, yellow crystals (from EtOH), m.p. 148 °C; IR (KBr): *v* 1722 (C=O), 3166, 3340 (2NH) cm^−1^; ^1^H-NMR (DMSO-*d*_6_): δ 2.58 (s, 3H, CH_3_), 7.12–7.98 (m, 15H, Ar–H), 9.33 (s, 1H, NH), 11.60 (s, 1H, NH); MS *m/z* (%): 368 (M^+^, 23), 248 (44), 167 (37), 104 (49), 77 (75), 60 (100); Anal. Calcd for C_23_H_20_ON_4_O (368.16): C, 74.98; H, 5.74; N, 15.21%. Found: C, 74.11; H, 5.67; N, 15.14%.

*Synthesis of 1-(5-oxo-4,4-diphenyl-4,5-dihydro-1*H*-imidazol-2-yl)-3-phenyl-1*H*-pyrazole-4-carb-aldehyde* (**20**). Dimethylformamide (2.19 g, 30 mmol) was cooled to below 5 °C and POCl_3_ (4.59 g, 30 mmole) was added dropwise under stirring for 30 min, to this mixture (2.68 g, 10 mmol) of compound **2** was added. The resulting mixture was refluxed for 2 h on water-bath. The precipitate obtained by pouring into ice-cold water was collected by filtration and recrystallized from absolute ethanol. Yield 80%, yellow crystals (from EtOH-dioxane), m.p. 294 °C; IR (KBr): *v* 1681, 1724 (2C=O), 3166 (NH) cm^−1^; ^1^H-NMR (DMSO-*d*_6_): δ 7.12–7.98 (m, 16H, Ar–H and 1H, pyrazolyl–H), 9.33 (s, 1H, NH), 9.88 (s, 1H, CHO); MS *m/z* (%): 406 (M^+^, 24), 248 (44), 182 (100), 104 (98), 77 (90), 51 (66); Anal. Calcd for C_25_H_18_N_4_O_2_ (406.14): C, 73.88; H, 4.46; N, 13.78%. Found: C, 73.95; H, 4.22; N, 13.72%.

*Synthesis of 5-amino-1-(5-oxo-4,4-diphenyl-4,5-dihydro-1*H*-imidazol-2-yl)-1*H*-pyrazole-4-carbonitrile* (**21**). A mixture of **2** (2.64 g, 10 mmol) and ethoxymethylenemalononitrile **17** (1.22 g, 10 mmol) in absolute ethanol (50 mL) was heated under reflux for 30 min. The solvent was evaporated under vacuum and the residual solid was crystallized from EtOH to give **21**. Yield 80%, yellow solid, m.p. 228 °C; IR (KBr): *v* 1724 (C=O), 2240 (CN), 3168 (NH), 3294, 3382 (NH_2_) cm^−1^; ^1^H-NMR (DMSO-*d*_6_): δ 6.68 (s, 2H, NH_2_), 7.12–7.98 (m, 11H, Ar–H and 1H, pyrazolyl–H), 9.33 (s, 1H, NH); MS *m/z* (%): 343 (M^+^+1, 5), 342 (M^+^, 14), 234 (39), 165 (68), 104 (65), 77 (100), 51 (71); Anal. Calcd for C_19_H_14_ N_6_O (342.12): C, 66.66; H, 4.12; N, 24.55%. Found: C, 66.40; H, 4.12; N, 24.43%.

*Synthesis of* *1-(5-oxo-4,4-diphenyl-4,5-dihydro-1*H*-imidazol-2-yl)-1*H*-pyrazolo[3,4-d]pyrimidin-4(7*H*)-one* (**22**). Compound **21** (10 mmol) in formic acid (20 mL, 85%) was refluxed for 3 h, cooled, poured onto ice-water to give a precipitate, which was filtered off, dried and recrystallized from EtOH to afford **22**. Yield 80%, yellow crystals (from EtOH-dioxane), mp 284 °C; IR (KBr): *v* 1671, 1723 (2C=O), 3166, 3280 (2NH) cm^−1^; ^1^H-NMR (DMSO-*d*_6_): δ 7.12–7.98 (m, 12H, Ar–H and 2H, pyrazolyl–H and pyrimidine–H), 9.33 (s, 1H, NH), 11.83 (s, 1H, NH); MS *m/z* (%): 470 (M^+^, 28), 323 (26), 208 (21), 165 (49), 93 (14), 77 (100); Anal. Calcd for C_20_H_14_N_6_O_2_ (370.12): C, 64.86; H, 3.81; N, 22.69%. Found: C, 64.60; H, 3.88; N, 22.60%.

*Synthesis of 2-(3,5-diphenyl-4,5-dihydro-1*H*-pyrazol-1-yl)-4,4-diphenyl-1*H*-imidazol-5(4*H*)-one* (**23**). A mixture of compound **2** (0.271 g, 1 mmol) and 3-(phenyl)-1-phenylprop-2-en-1-one (**18**, 0.238 g, 1 mmol) in acetic acid (20 mL) was refluxed for 7 h. Excess of solvent was removed under reduced pressure and the reaction mixture was added to crushed ice. The product separated was filtered, washed with water, dried and recrystallized from DMF. Yield 80%, yellow crystals (from EtOH-dioxane), m.p.141 °C; IR (KBr): *v* 1722 (C=O), 3169 (NH) cm^−1^; ^1^H-NMR (DMSO-*d*_6_): δ 2.91 (dd, 1H, H_A_, *J* = 17.2, 6.5 Hz), 4.14 (dd, 1H, H_B_, *J* = 17.3, 12.6 Hz), 5.98 (dd, 1H, H_X_, *J* = 12.8, 6.5 Hz), 7.12–7.98 (m, 20H, Ar–H), 9.35 (s, 1H, NH); MS *m/z* (%): 456 (M^+^, 10), 357 (12), 248 (46), 181 (73), 104 (93), 77 (76), 51 (66); Anal. Calcd for C_30_H_24_N_4_O (456.20): C, 78.92; H, 5.30; N, 12.27%. Found: C, 78.84; H, 5.34; N, 12.22%.

### 3.5. Preliminary Antimicrobial Screening

A selection of the prepared compounds (namely **6a**, **6c**, **6f**, **6h**, **12**, **13**, **14**, **15**, **20**, **22** and **23**) were screened for their antibacterial activity (in nutrient agar broth) and antifungal activity (in Dox’s medium and Saboured’s agar) by the agar diffusion method [37,38] at a concentration 20 mg/mL using DMSO as solvent and blank. 

## 4. Conclusions

We have established a new and efficient synthesis of a novel series of 7,7-diphenyl-1,2-dihydroimidazo[2,1-*c*][1,2,4]triazin-6(7*H*)-ones. We could also extend this technique to the synthesis of new pyrazole containing imidazolone moieties. The antifungal and antibacterial activities of the newly synthesized compounds were evaluated.
